# The Modulatory Properties of *Astragalus membranaceus* Treatment on Triple-Negative Breast Cancer: An Integrated Pharmacological Method

**DOI:** 10.3389/fphar.2019.01171

**Published:** 2019-10-14

**Authors:** Cun Liu, Kejia Wang, Jing Zhuang, Chundi Gao, Huayao Li, Lijuan Liu, Fubin Feng, Chao Zhou, Kang Yao, Laijun Deng, Lu Wang, Jia Li, Changgang Sun

**Affiliations:** ^1^First School of Clinical Medicine, Shandong University of Traditional Chinese Medicine, Jinan, China; ^2^Department of Basic Medical Sciences, School of Medicine, Xiamen University, Xiamen, China; ^3^Department of Oncology, Weifang Chinese Medicine Hospital, Weifang, China; ^4^College of Traditional Chinese Medicine, Shandong University of Traditional Chinese Medicine, Jinan, China; ^5^College of Basic Medicine, Weifang Medical University, Weifang, China; ^6^Department of Basic Medical Science, Qingdao University, Qingdao, China; ^7^Department of Oncology, Affiliated Hospital of Shandong University of Traditional Chinese Medicine, Jinan, China

**Keywords:** triple-negative breast cancer, *Astragalus membranaceus*, integrated pharmacology, *Astragalus polysaccharides*, PIK3CG/AKT/BCL2 pathway

## Abstract

**Background:** Studies have shown that the natural products of *Astragalus membranaceus* (AM) can effectively interfere with a variety of cancers, but their mechanism of action on breast cancer remains unclear. Triple-negative breast cancer (TNBC) is associated with a severely poor prognosis due to its invasive phenotype and lack of biomarker-driven-targeted therapies. In this study, the potential mechanism of the target composition acting on TNBC was explored by integrated pharmacological models and *in vitro* experiments.

**Materials and Methods:** Based on the Gene Expression Omnibus (GEO) database and the relational database of Traditional Chinese Medicines (TCMs), the drug and target components were initially screened to construct a common network module, and multiattribute analysis was then used to characterize the network and obtain key drug-target information. Furthermore, network topology analysis was used to characterize the betweenness and closeness of key hubs in the network. Molecular docking was used to evaluate the affinity between compounds and targets and obtain accurate combination models. Finally, *in vitro* experiments verified the key component targets. The cell counting kit-8 (CCK-8) assay, invasion assay, and flow cytometric analysis were used to assess cell viability, invasiveness, and apoptosis, respectively, after *Astragalus polysaccharides* (APS) intervention. We also performed western blot analysis of key proteins to probe the mechanisms of correlated signaling pathways.

**Results:** We constructed “compound-target” (339 nodes and 695 edges) and “compound-disease” (414 nodes and 6458 edges) networks using interaction data. Topology analysis and molecular docking were used as secondary screens to identify key hubs of the network. Finally, the key component APS and biomarkers PIK3CG, AKT, and BCL2 were identified. The *in vitro* experimental results confirmed that APS can effectively inhibit TNBC cell activity, reduce invasion, promote apoptosis, and then counteract TNBC symptoms in a dose-dependent manner, most likely by inhibiting the PIK3CG/AKT/BCL2 pathway.

**Conclusion:** This study provides a rational approach to discovering compounds with a polypharmacology-based therapeutic value. Our data established that APS intervenes with TNBC cell invasion, proliferation, and apoptosis *via* the PIK3CG/AKT/BCL2 pathway and could thus offer a promising therapeutic strategy for TNBC.

## Introduction

Triple-negative breast cancer (TNBC) accounts for 10–20% of all breast tumors and has a higher degree of malignancy and a worse prognosis than other breast cancer subtypes (Bianchini et al., 2016; Logue et al., 2018). Although research has provided insight into its pathogenesis and treatment strategies, the incidence and mortality of TNBC remain high (Ghoncheh et al., 2016; Viens et al., 2017). The side effects and drug resistance of conventional drug therapy seriously limit their therapeutic effects on TNBC (Chang et al., 2018; Kim et al., 2018a). Therefore, research to determine more effective and safer drugs to improve the prognosis and survival of TNBC patients is needed.

With arsenicals leading to significant breakthroughs in the field of leukemia treatment (Zhu et al., 1997), the unique antitumor activity of many natural products has attracted increasing attention. *Astragalus membranaceus* (AM), which is rich in flavonoids, saponins, and polysaccharides, has been widely used in cancer treatment in recent years (Wang et al., 2014; Zhu et al., 2015; Kong et al., 2018). For example, quercetin, formononetin, calycosin, etc. have shown broad antitumor activity (Gao et al., 2014; Massi et al., 2017; Kim et al., 2018b; Rauf et al., 2018). The diverse composition of AM is the material basis of its effect; however, this diversity also complicates pharmacological research. Although some progress has been made in the identification of natural compound targets, limitations still exist in studies that are based on only known effector proteins and approved drugs, such as high late-stage clinical attrition rates, cumbersome *post hoc* deconvolution, and low efficiency and innovativeness (Terstappen et al., 2007; Hutchinson and Kirk, 2011; Waring et al., 2015). Therefore, it is necessary to find an effective innovative measure to elucidate the multiple target mechanisms of natural compounds and thus better understand their phenotypic effects.

The “integrated pharmacology” (IP) method derived from traditional research is becoming increasingly popular (Li et al., 2018b; Tabrizi et al., 2018). IP aims to find active substances that can intervene in underlying impaired mechanisms and deregulated interactions by modulating the activity of several hubs or by targeting multiple pathways in complex disease networks (Kitano, 2007). There is great potential to address initial hypotheses for both *in vitro* and *in vivo* target validation studies that rely on computational methods. In particular, with innovation in microarray technologies and the construction of public repositories of microarray data, the characterization of transcriptome profiles may change at an unprecedented ways (Butte, 2002; Lu et al., 2009). Pharmacological research has also been improved unprecedentedly with the assistance of computer technology (Xu et al., 2017). The development of network pharmacology would enable effective elucidating of not yet explored natural products, hence providing systematic methods to extend the druggable space in various complex diseases (Kibble et al., 2015). By the network construction and analysis of multiobjective active components and key targets, interactions between drugs and specific nodes or modules can be elucidated to better identify potential mechanisms.

**Figure 1:**
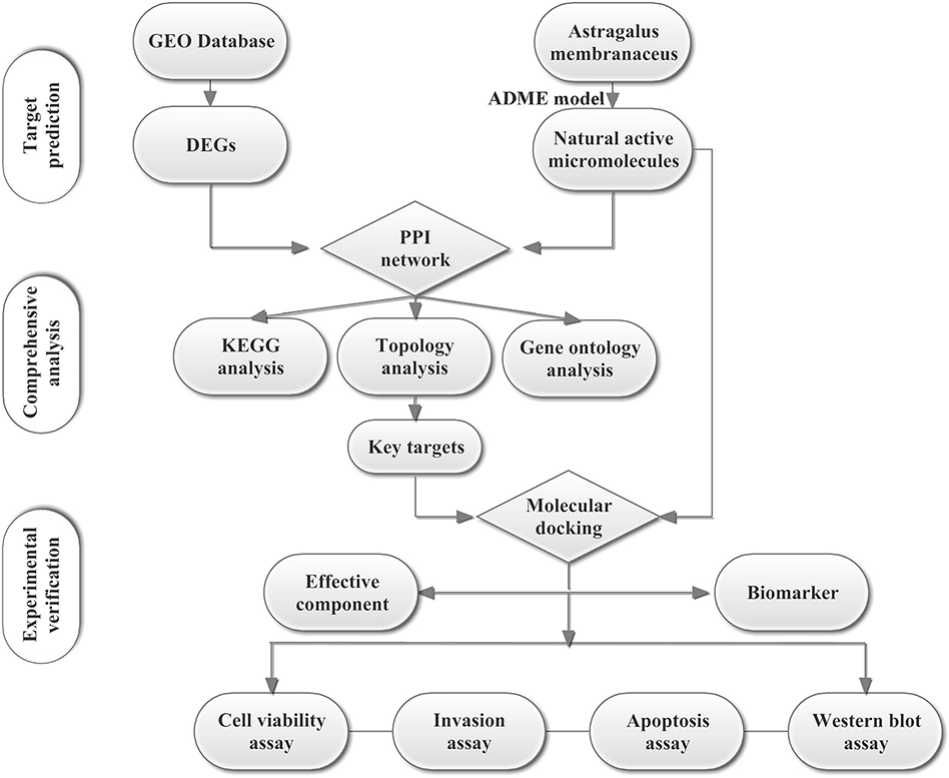
Scheme for the integrated pharmacology approach.

To explore the possible and novel interference mechanisms of AM in TNBC, we constructed a new IP model. This model combines microarray data, pharmacokinetic screening, multilevel network construction, and *in vitro* experiments. The scheme for the model is shown in **Figure 1**. We propose that *Astragalus polysaccharides* (APS) could target the PIK3CG/AKT/BCL2 signaling pathway and then influence TNBC. This study provides a rational way to screen effective compounds and targets, which enhances our ability to identify active molecules intervene complex disease.

## Materials and Methods

### Differentially Expressed Gene Search, Identification, and Analysis

We downloaded the microarray data GSE38959, GSE65194, and GSE76275 from the Gene Expression Omnibus (GEO) database (http://www.ncbi.nlm.nih.gov/geo/) using their microarray platform as GPL4133 and GPL570 (Affymetrix Human Gene Expression Array). GSE38959 aims to compare gene expression changes between TNBC and normal mammary ductal tissues, while the latter two datasets focus on comparing TNBC with hormone receptor-positive breast cancer. Their intersection can be considered as unique biomarkers of TNBC. Differentially expressed genes (DEGs) were screened using the online tool GEO2R/R package limma. Limma package can fit a linear model for the expression of each gene and then carry out residual analysis to obtain the appropriate t statistics, which makes the analysis results more reliable. In the present study, P < 0.05 and |log2 fold change (FC)| > 1 were set as the cut-off criteria between three groups. Venn diagrams were used to further calculate the intersection of DEGs from two GEO microarray datasets. The heatmap of overlapping DEGs was obtained after subtracting Pearson correlation clustering using online Morpheus software (https://software.broadinstitute.org/morpheus).

### Database Construction and ADME Screening of AM

All the ingredients of AM were manually collected from the Traditional Chinese Medicine Systems Pharmacology Database (TCMSP, http://ibts.hkbu.edu.hk/LSP/tcmsp.php) and TCM Database@Taiwan (http://tcm.cmu.edu.tw/). An *in silico* integrative model, ADME (absorption, distribution, metabolism, and excretion), was used to predict the pharmacokinetic behaviors of chemical compounds. In this process, three ADME-related models, including oral bioavailability (OB), Caco-2 permeability, and drug likeness (DL), were employed to identify the bioactive compounds with favorable pharmacokinetic properties. As one of the most vital pharmacokinetic parameters of orally administered drugs, OB plays a key role in the efficiencies of bioactive compounds in systemic circulation (Saghir, 2009; Tian et al., 2011; Xu et al., 2012). The Caco-2 cell model was used as a standard *in vitro* screening tool for predicting intestinal drug absorption and drug transport mechanisms. Drug likeness is a qualitative profile used in drug design for how “drug-like” a substance is with respect to factors such as bioavailability. The DL of compounds in AM was assessed based on molecular descriptors and the Tanimoto coefficient (Ru et al., 2014),

$$T\left(A,B\right)=\frac{A*B}{A^{2}+B^{2}-A*B}$$

where A represents any ingredient descriptor of AM, and B is the average of this characteristic over all molecules in the DrugBank database. In this study, the ingredients meeting the criteria of OB ≥ 30%, Caco-2 > −0.4, and DL ≥ 0.18 were chosen for further research. In addition, we manually retrieved articles published by PubMed (https://www.ncbi.nlm.nih.gov/pubmed). In this step, we searched the PubMed database with *Astragalus propinquus* as the subject (mesh word) and *A. membranaceus* and *Astragalus mongholicus* as free words. The search strategy was set as {[(*A. membranaceus*) OR *A. mongholicus*)] OR “*A. propinquus*”[Mesh]}. Further, we manually screened the articles and incorporated the components that were significant and clearly identified in previous studies.

### Identification of Associated Compound Targets

Protein information for active ingredients was obtained by using the Traditional Chinese Medicine Systems Pharmacology (TCMSP) database (http://lsp.nwu.edu.cn/tcmsp.php), SwissTargetPrediction (http://www.swisstargetprediction.ch/), and the PharmMapper database (http://lilab.ecust.edu.cn/pharmmapper/index.php), which all predict interactions between compounds and target proteins through structural fitness and previous studies. The SMILES of each component was obtained from PubChem (http://pubchem.ncbi.nlm.nih.gov) and then imported into SwissTargetPrediction. Additionally, after the molecular structure was obtained from PubChem, it was transformed into Open Bable and saved as a “mol2” file, which is suitable for PharmMapper. Accurate information on genes and proteins was further extracted from UniProtKB (http://www.uniprot.org/), which is a protein database with abundant and consistent annotation.

### Protein–Protein Interaction Network Construction and Analysis

We attempted to characterize the multicomponent treatment features of AM and to explore the biological effects of small molecular compounds. Two networks, including the “compound-target” network of AM and the “compound-disease” network, were constructed using the STRING database (https://string-db.org/). Interaction data were further read in Cytoscape software for visualization and bioinformatics analysis. Gene Ontology (GO) and pathway enrichment analyses were based on the ClueGO plugin and DAVID database (https://david.ncifcrf.gov/) to facilitate the biological interpretations. Finally, we used the CentiScaPe plug-in to implement topological attribute analysis and represent the key hubs in the network based on the connectivity of each node.

### Molecular Dynamic Simulation Docking and Target Fishing

To investigate the interaction relationship and mechanisms of action between candidate compounds and targets in depth, we conducted a molecular dynamics study (molecular docking and simulation score) of the two based on the Surflex-docking module in SYBYL. It creates a prototype molecule with a representative protein activity docking pocket and uses hydrogen bond donors, receptors, and water molecules to populate the protein docking pockets. SYBYL is also known for its high-accuracy docking, high true-positive rate, and fast speed. Before docking, the X-ray crystal structures of DEGs were extracted from the RCSB Protein Data Bank (http://www.rcsb.org/). Then, they were imported into SYBYL to remove cocrystallized ligand and structural water molecules and adjunct polar hydrogen atoms. Ligand preparation was based on ChemDraw Ultra 8.0 software, which was used to construct two-dimensional structures that were saved in the mol2 format. Receptor and ligand molecular docking was assessed based on the Surflex-docking module. The active pocket was obtained based on the ligand mode, and the default values for the ProtoMol bloat and ProtoMol threshold parameters were set as 0 and 0.50 Å, respectively. Hydrogen bond strength and domain structure similarity were used to evaluate the stability and reliability of docking. The differential expression of key genes in different tissues was characterized by the Gene Expression Profiling Interactive Analysis (GEPIA) database (http://gepia.cancer-pku.cn). We used log2 (TPM + 1) for log-scale, and the jitter size was set to 0.4.

### Cell Culture

The human TNBC cell line MDA-MB-231 (estrogen receptor negative, progesterone receptor negative, and human epidermal growth factor receptor-2 negative) was obtained from iCell Bioscience Inc. (Shanghai, China) and grown in Dulbecco’s Modified Eagle’s Medium (Invitrogen, Carlsbad, CA, USA) supplemented with 10% fetal bovine serum (FBS, Gibco, Gaithersburg, MD, USA) and 1% penicillin/streptomycin (Gibco). All cells were grown as monolayers and maintained in a cell culture incubator at 37°C and 5% CO2.

### Cell Viability Assay

The cell counting kit-8 (CCK-8, Dojindo, Kumamoto, Japan) colorimetric assay was performed to assess cell proliferation. MDA-MB-231 cells were seeded (1×10^3^ cells/well) into 96-well microplates and allowed to adhere overnight. A total of three repeat wells were seeded for each group. The medium was removed the following day, and fresh medium with APS (98% purity, Yingxin Laboratory, Shanghai, China) (0, 0.25, 0.5, 1, or 2 mg/ml) was added. Ten microliters of CCK-8 reagent was added to each well at the indicated time points (1, 2, 3, and 4 days after seeding into plates), and the mixture was incubated for an additional 2 h. The absorbance was measured using a Benchmark Microplate Reader (Bio-Rad Laboratories, Hercules, CA, USA) at 450 nm.

### Invasion Assay

Serum-free cell culture medium was added to the upper chamber of a 24-well transwell plate, and the cell density was adjusted to 1 × 10^4^ cells/ml. MDA-MB-231 cells (100 μl) were seeded into the upper chamber of the Transwell migration chamber and incubated with different concentrations of APS (0, 0.25, 0.5 mg/ml) for 24 h. The cells were fixed with methanol for 20 min and stained with 0.1% crystal violet for 5 min, the chamber was then placed under an inverted microscope, and the remaining cells were counted.

### Apoptosis Assay

Apoptosis was evaluated using an Annexin-V fluorescein isothiocyanate (FITC) Apoptosis Kit (Solarbio Life Sciences, Beijing, China) according to the manufacturer’s protocol. Briefly, cells were treated with 1 mg/ml APS for 72 hours. Next, 2.0 ml of annexin V-FITC stain and 10 ml of propidium iodide (PI) stain were added to 500 μl of binding buffer and mixed, which is followed by incubation at room temperature in the dark for 15 min. Finally, apoptosis was evaluated by flow cytometry.

### Western Blot Assays

First, MDA-MB-231 cells were treated in triplicate with or without APS at concentrations of 0.25 and 0.5 mg/ml for 48 h. Then, cells were lysed on ice with mixed RIPA buffer containing 150 mM NaCl, 1% Triton X-100, 5 mM ethylenediaminetetraacetic acid, 10 mM NaF, 1×protease inhibitors, and PMSF (Invitrogen, Carlsbad, CA, USA). Proteins (30 μg) were loaded onto an SDS-polyacrylamide gel electrophoresis (PAGE) gel and transferred to a nitrocellulose membrane (Millipore, Bedford, MA). The membrane was blocked with 5% nonfat milk for 1 h at room temperature and incubated with primary antibodies against PIK3CG, AKT, p-AKT, and BCL2 (1:1000, Cell Signaling Technology, Inc. Beverly, MA, USA) overnight at 4°C. The next day, after washing with cold TBST, the membranes were incubated with appropriate horseradish peroxidase-conjugated secondary antibodies for 1 h at room temperature. Finally, the bands were visualized with the ChemiDoc XRS System (Bio-Rad, Hercules, CA, USA).

### Statistical Analysis

Data are expressed as the mean ± standard deviation of at least three independent experiments. Quantitative analysis of figures was carried out using the GraphPad Prism 6.0 statistical software package (GraphPad Software Inc., La Jolla, CA). The significance of multiple comparisons was determined by one-way analysis of variance (ANOVA) with SPSS, version 13.0 (SPSS, Inc., Chicago, IL, USA). P < 0.05 was considered to indicate a statistically significant difference.

## Results

### Deg Identification

**Figure 2:**
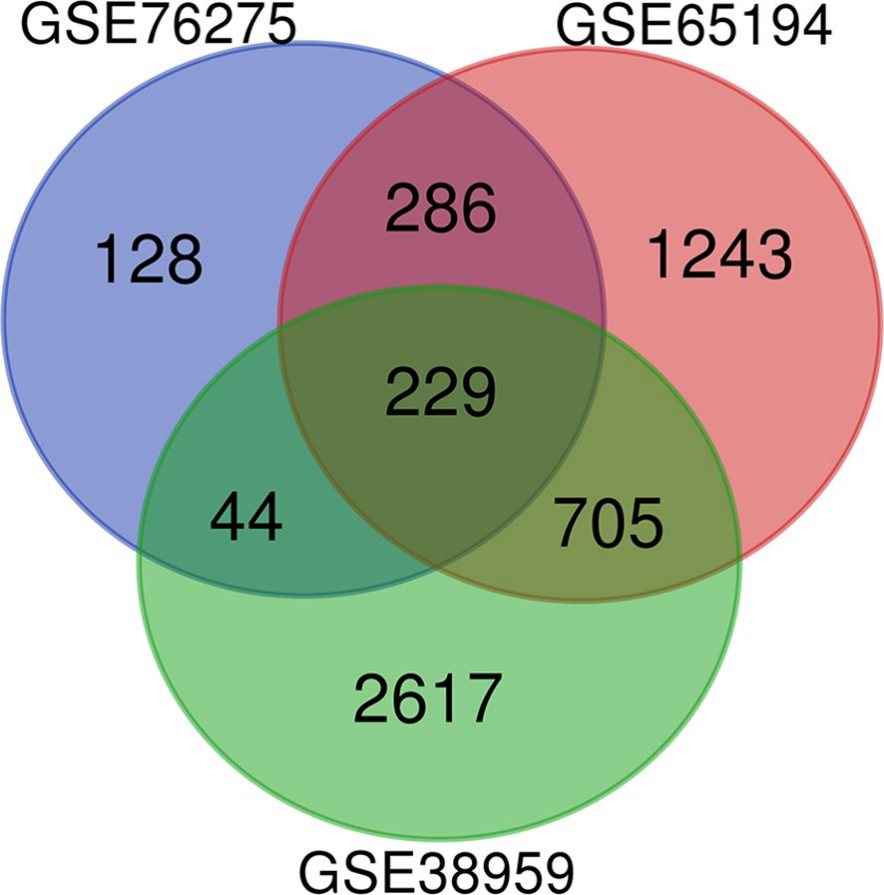
DEGs between TNBC were identified by three transcription profile datasets (GSE38959, GSE65194, GSE76275). DEGs were identified with the classical t test, and statistically significant DEGs were defined using P < 0.05 and |log2 fold change (FC)| > 1 as the cutoff criteria.

**Figure 3:**
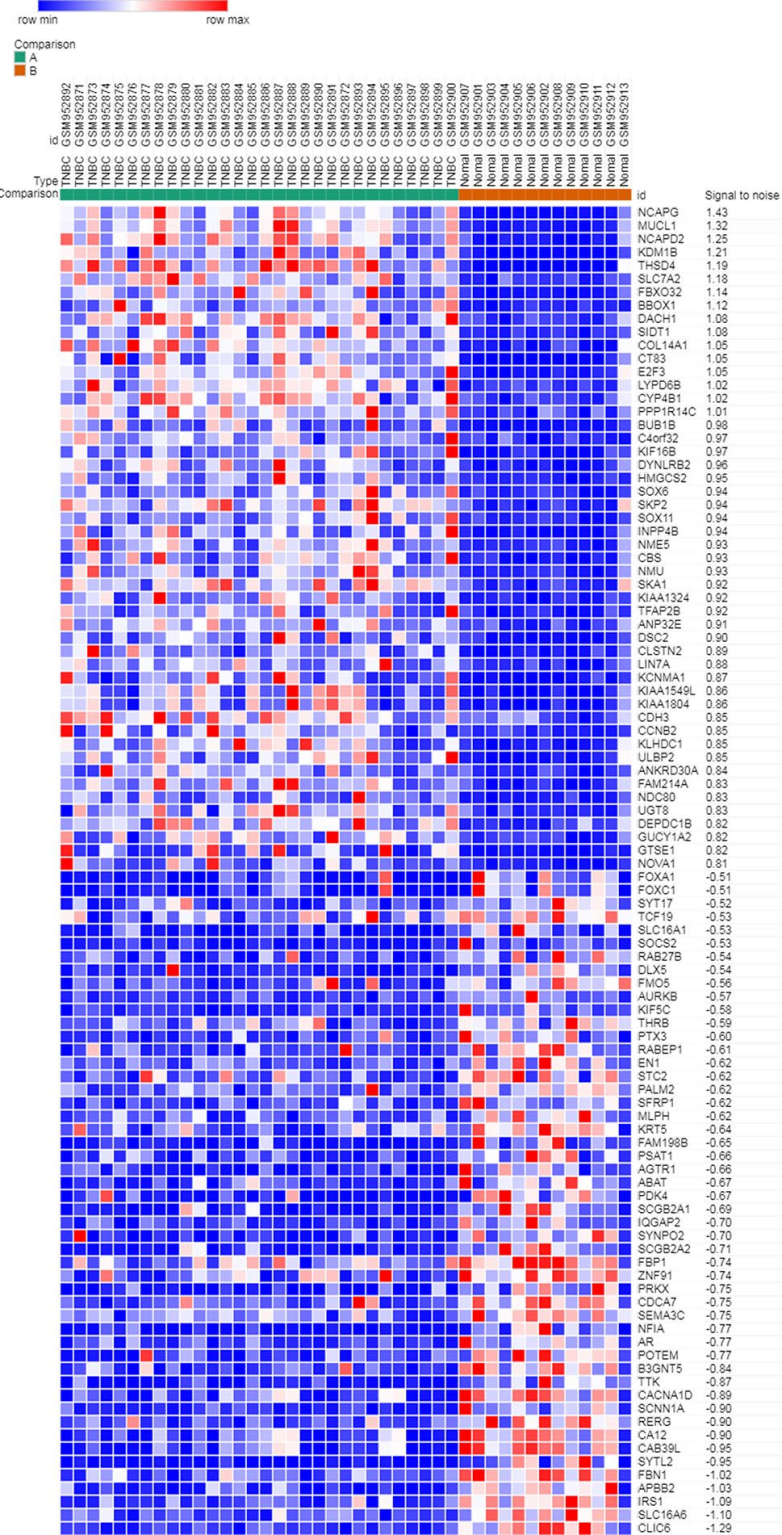
Heatmap plot of the top 50 DEGs between tumors and normal tissues. The heatmap was obtained after subtracting Pearson correlation clustering, where red represents higher gene expression levels, and blue represents lower gene expression levels.

Microarray expression data, no. GSE38959, no. GSE65194, and no. GSE76275, were obtained from the GEO database and reanalyzed. We focused on coexpression analysis of DEGs, which is more sensitive for identifying treatment-induced deregulation among interacting genes. A Venn diagram showed the overlap of a total of 229 significant targets (**Figure 2**). The heatmap was structured after clustering the top 50 overlapping DEGs with the highest |log2FC| values (**Figure 3**). The results showed a significant difference in the DEG expression among the three samples.

### Screening for Active Compounds and Relevant Targets

Because of the multicomponent characteristics of herbal medicines, it is critical to select drugs with satisfactory pharmacokinetic properties for further research. A total of 87 compounds from AM were obtained from the TCMSP database, and 16 active components were selected based on the ADME model. Further, a total of 1,088 studies related to *Astragalus* were found through literature search. After careful screening, we found that, in addition to the 16 compounds screened by the ADME model, APS (192 studies) and astragaloside IV (170 studies) appeared most frequently. And both of them showed real and extensive biological activity in previous studies. Thus, 18 active compounds of AM were ultimately chosen for further investigation, detailed data was listed in **Table S1**. Compound-related genes were obtained from the TCMSP, SwissTargetPrediction, and PharmMapper databases, and the remaining 310 genes were obtained after deletion of duplicates; raw data and final results were shown in **Tables S2** and **S3**, respectively. Their official names were obtained from the UniProtKB database and used to build networks for further characterization of biological characteristics.

### Network Construction and Preliminary Screening of Key Targets

**Figure 4:**
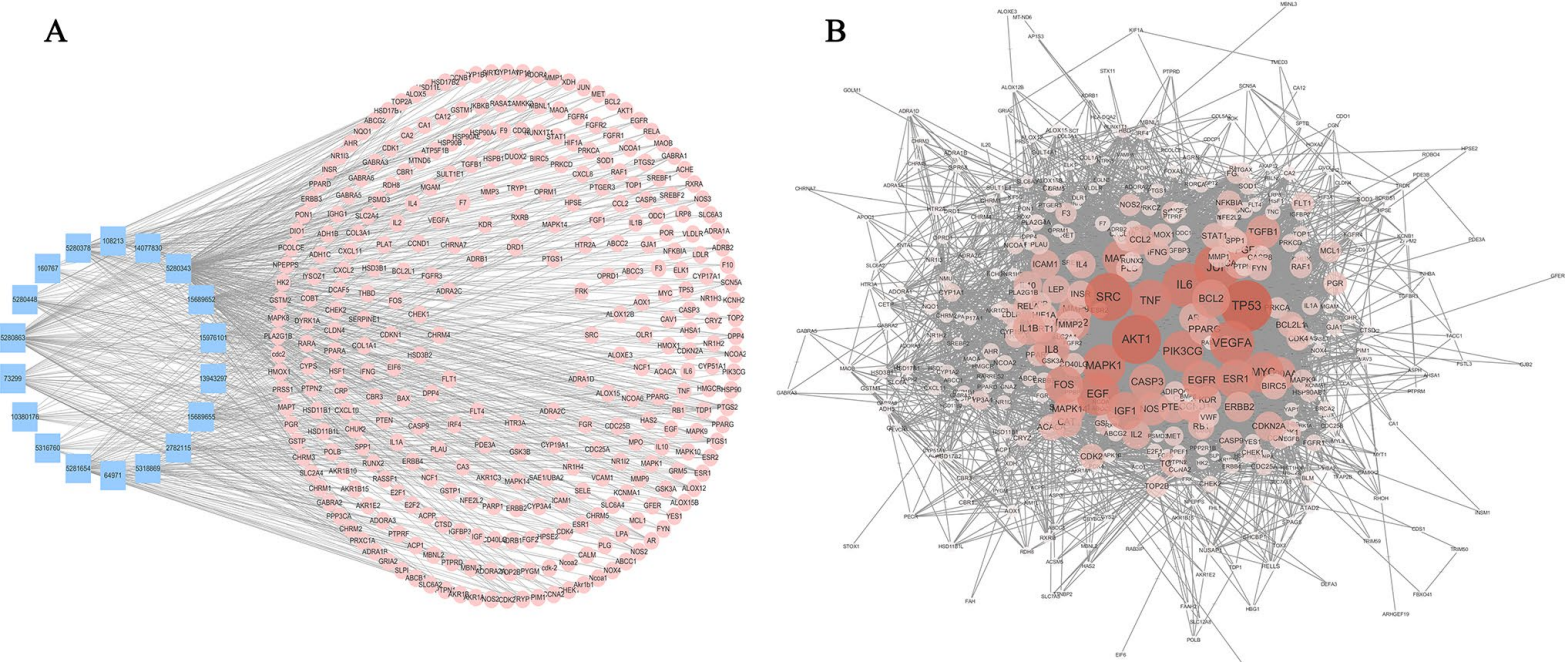
Compound-target network and compound-disease network constructed by STRING and Cytoscape. **(A)** Compound-target network: the blue rectangles represent the small molecular component in AM, and the numbers in the rectangles depict the PubChem IDs. The pink circles represent the relevant targets. **(B)** Compound-disease network: as the color deepens and the circle increases, the connectivity of the targets is greater, and the degree of criticality is higher.

Bioactive natural compounds can regulate a network by binding multiple hubs, which implies that they are multi-targeted when intervening diseases. Hence, to predict the target information for the active constituents of AM, two networks, “compound-target” (C-T) (**Figure 4A**) and “compound-disease” (C-D) (**Figure 4B**), were constructed to analyze the mechanism.

**Figure 5:**
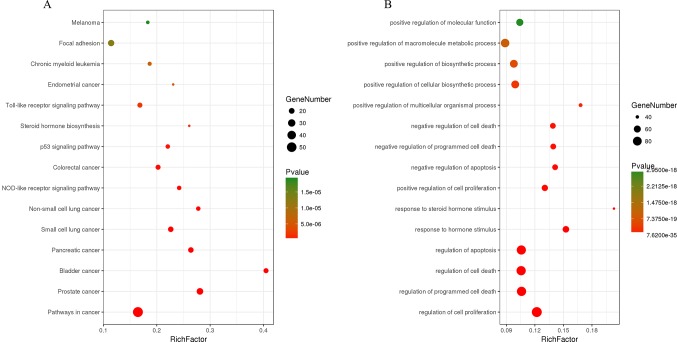
Enrichment of KEGG pathways **(A)** and gene ontology biological process terms **(B)** for DEGs. P < 0.05 and false discovery rate (FDR) < 0.05 were set as the cutoff criteria; the top 15 meaningful terms are presented in the bubble chart.

The C-T network consisted of 339 nodes and 695 edges in total. Pathway enrichment (**Figure 5A**) analysis and GO analysis (**Figure 5B**) were further performed to clarify the biological functions of major natural compounds of AM. The results showed that the most representative GO biological process (BP) terms centered on the “regulation of cell proliferation,” “regulation of apoptosis,” “regulation of programmed cell death,” “regulation of cell death,” “negative regulation of apoptosis,” “negative regulation of programmed cell death,” “negative regulation of cell death,” and “positive regulation of cell proliferation,” which indicate the well-documented pharmacological and biological effects on cell proliferation and apoptosis. Additionally, the enriched pathways included the “p53 signaling pathway,” “toll-like receptor signaling pathway,” “MAPK signaling pathway,” “apoptosis,” “VEGF signaling pathway,” and a variety of tumor-related pathways, such as “prostate cancer,” “bladder cancer,” “pancreatic cancer,” and “small cell lung cancer.” This result comprehensively shows that AM has good antitumor activity and validates the reliability of the prediction target.

**Figure 6:**
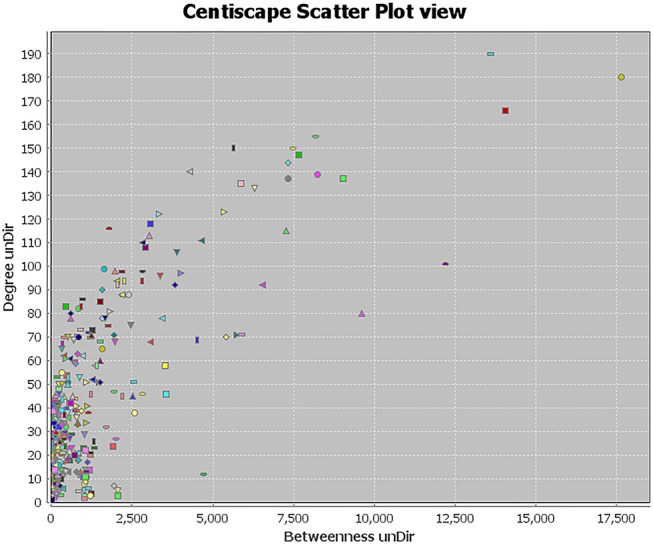
Topological attribute analysis diagram of the compound-disease network. CentiScaPe plug-in in Cytoscape was used to read network data and performed topology analysis based on connectivity between nodes. The horizontal axis represents betweenness, and the ordinate represents the connectivity degree. The graphic in the table represents each target in the network.

Topology attribute analysis describes the characteristics of the C-D network (414 nodes and 6,458 edges) based on degree and betweenness (**Figure 6**). The results showed that, with a higher connectivity of nodes in the network, there were fewer corresponding nodes. The cutoff value was set to twice the median degree, and 15 key genes based on this value were obtained (**Table 1**).

**Table 1:** Key genes obtained by topological attribute analysis.

ID	Gene	Degree	Betweenness	Closeness
1	TP53	190	17637.654	0.57032967
2	AKT1	180	14043.39	0.57411504
3	SRC	166	13610.389	0.55212766
4	JUN	155	12211.12	0.5672131
5	VEGFA	150	9618.002	0.5372671
6	IL6	150	9021.176	0.5492064
7	PIK3CG	147	8239.002	0.5356037
8	MAPK1	144	8187.938	0.54516804
9	MYC	140	7663.9834	0.5317623
10	TNF	139	7466.6	0.53450054
11	ESR1	137	7331.85	0.5328542
12	EGF	137	7313.31	0.536157
13	EGFR	135	7259.8237	0.5400624
14	BCL2	133	6536.154	0.5123396
15	FOS	123	6301.0967	0.51744765

### Docking Combination Screen

**Figure 7:**
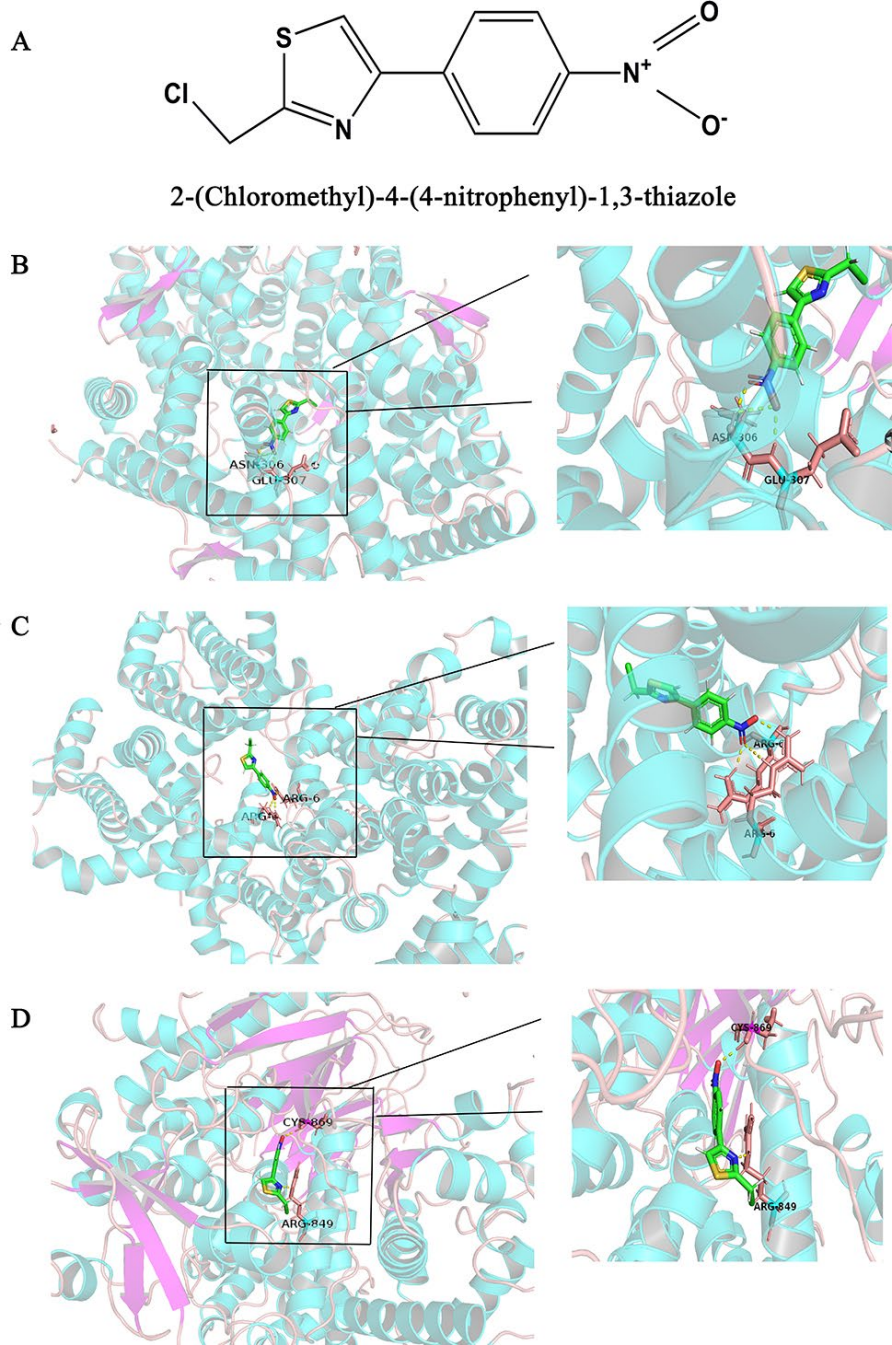
Binding interactions of APS with receptors. **(A)** Structural Formula of APS, **(B)** AKT (PDB ID: 3QKK; score: 4.9589), **(C)** BCL2 (PDB ID: 4AQ3; score: 4.4951), and **(D)** PIK3CG (PDB ID: 2CHX; score: 5.0091). The molecules are present as ball and stick models. The dotted yellow lines in these pictures represent H-bonds with distance units of Å.

**Figure 8:**
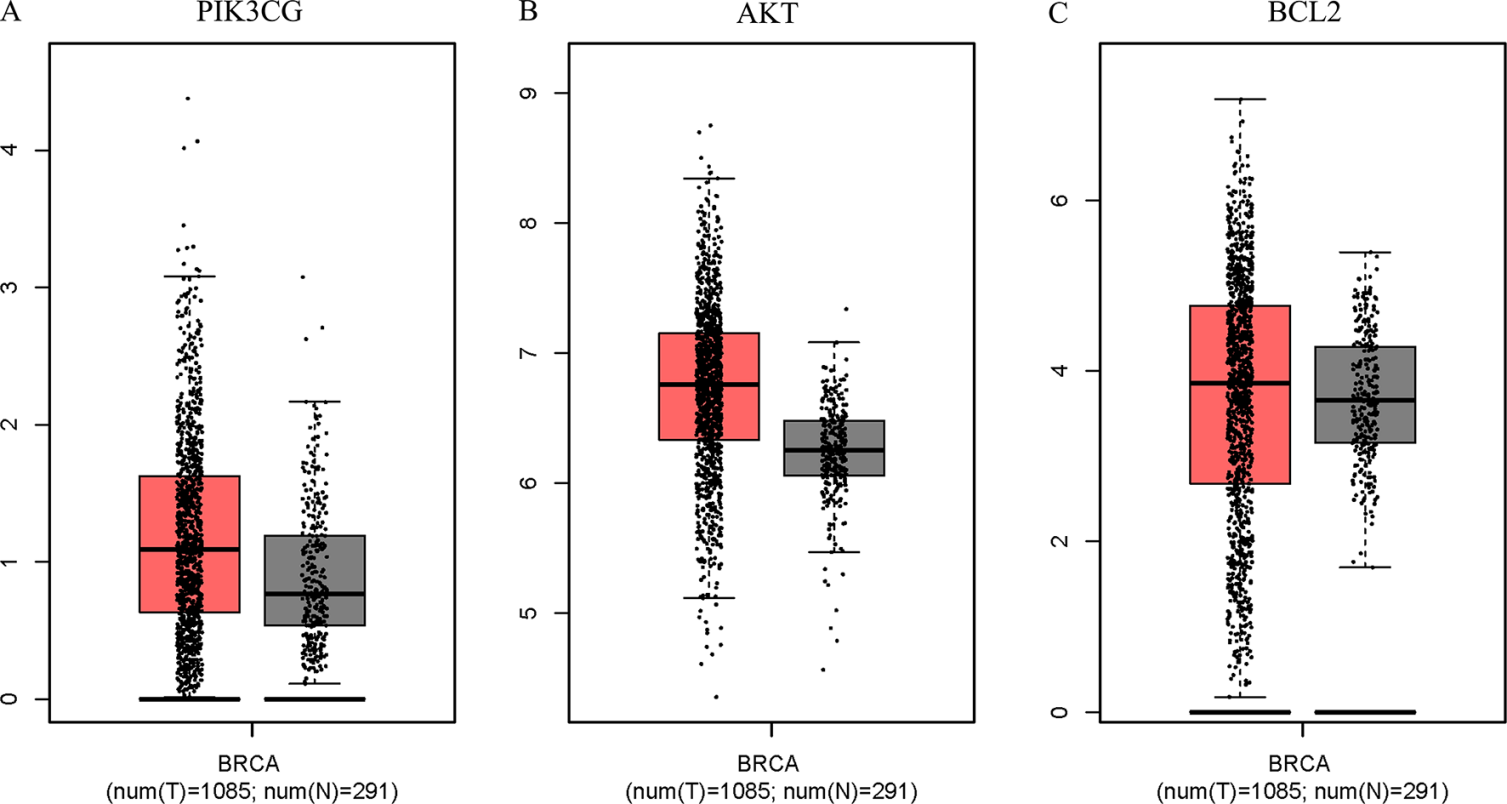
PIK3CG **(A)**, AKT **(B)**, and BCL2 **(C)** expression was upregulated in breast tumors compared with that in normal mammary tissues based on TCGA data. The total sample was 1,376, of which the number of tumor samples and normal samples were 1,085 and 291, respectively. The jitter size was set as 0.4, and we used log2 (TPM + 1) for log-scale.

The Surflex-docking score is based on the binding affinity and hydrogen bond strength between ligands and proteins (Ai et al., 2015). By docking interrelated natural compounds and proteins, we discovered good docking results between the high potential compound APS (**Figure 7A**) and the proteins AKT (PDB ID: 3QKK; score: 4.9589), BCL2 (PDB ID: 4AQ3; score: 4.4951), and PIK3CG (PDB ID: 2CHX; score: 5.0091) (**Figures 7B–D**). The differential expression of the three key genes was further displayed by boxplots based on the GEPIA database (**Figure 8**). These data provide theoretical evidence that APS in AM may play an important role in regulating TNBC. Due to the typical enrichment of PIK3CG, AKT, and BCL2 in the classical PI3K/AKT pathway, further *in vitro* experiments will expand our knowledge of PIK3CG/AKT/BCL2.

### APS Inhibits the Growth and Invasion of TNBC Cells

**Figure 9:**
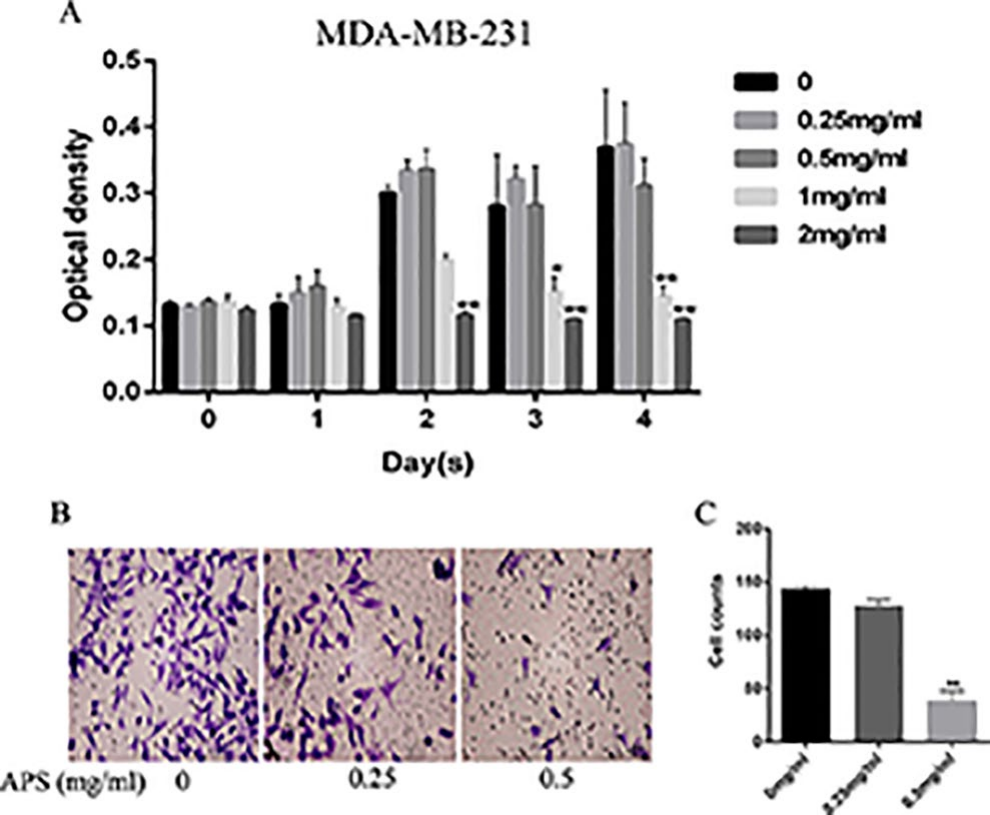
APS inhibits TNBC cell growth and invasion *in vitro*. **(A)** The optical density at the indicated concentrations of APS in MDA-MB-231 cells was detected by the CCK-8 assay. **(B–C)** Cells were cultured in triplicate with the indicated concentrations of APS for 24 h. The invaded cells were stained with crystal violet and counted under a light microscope. *p < 0.05, **p < 0.01 (compared with the 0 mg/ml group).

Antiproliferative effects were assessed by CCK-8 assays. Briefly, MDA-MB-231 cells were incubated with different concentrations of APS for 24, 48, 72, and 96 h; the concentrations were set to 0, 0.25, 0.5, 1, and 2 mg/ml, respectively. The results showed that the proliferation inhibition was concentration-dependent (**Figure 9A**). TNBC cells treated at a concentration of 2 mg/ml showed inhibited proliferation at 48 h. When cultured for 72 hours, this condition also began to appear in the 1 mg/ml dose group. The possibility of whether APS could inhibit the invasive ability of TNBC cells was also explored. After the cells were cultured with different concentrations of drug (0, 0.25, 0.5 mg/ml) for 24 h, the invasion ability of MDA-MB-231 cells showed varying degrees of decline that were concentration-independent (**Figures 9B, C**).

### Effects of APS on BC Cell Apoptosis

**Figure 10:**
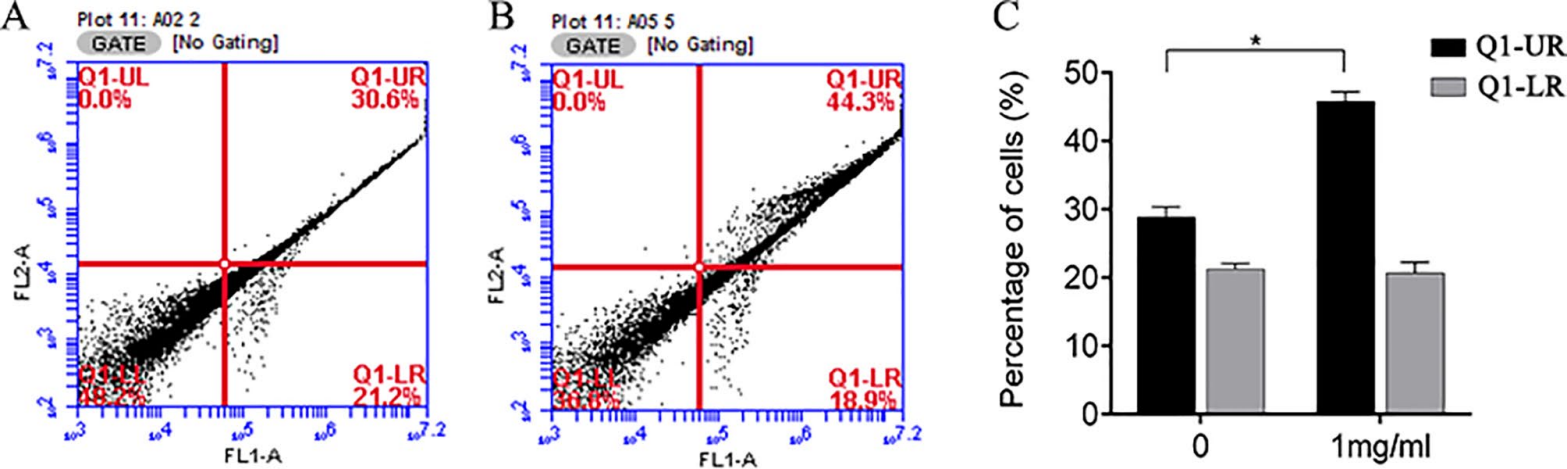
Flow cytometry was used to analyze apoptosis of MDA-MB-231 cells treated with APS. **(A–B)**: Control group and 1 mg/ml dose group. **(C)** The bar graphs represent the quantitative analysis of apoptotic cells. *p < 0.05.

We also investigated whether the suppression of cell growth by APS was due to an impact on cell apoptosis. The apoptosis of MDA-MB-231 cells was measured by flow cytometry. APS was found to affect late apoptosis at a dose of 1 mg/ml; however, the rate of early apoptosis appeared relatively unaffected (**Figure 10**).

### Effect of APS On Protein Expression In TNBC Cells

**Figure 11:**
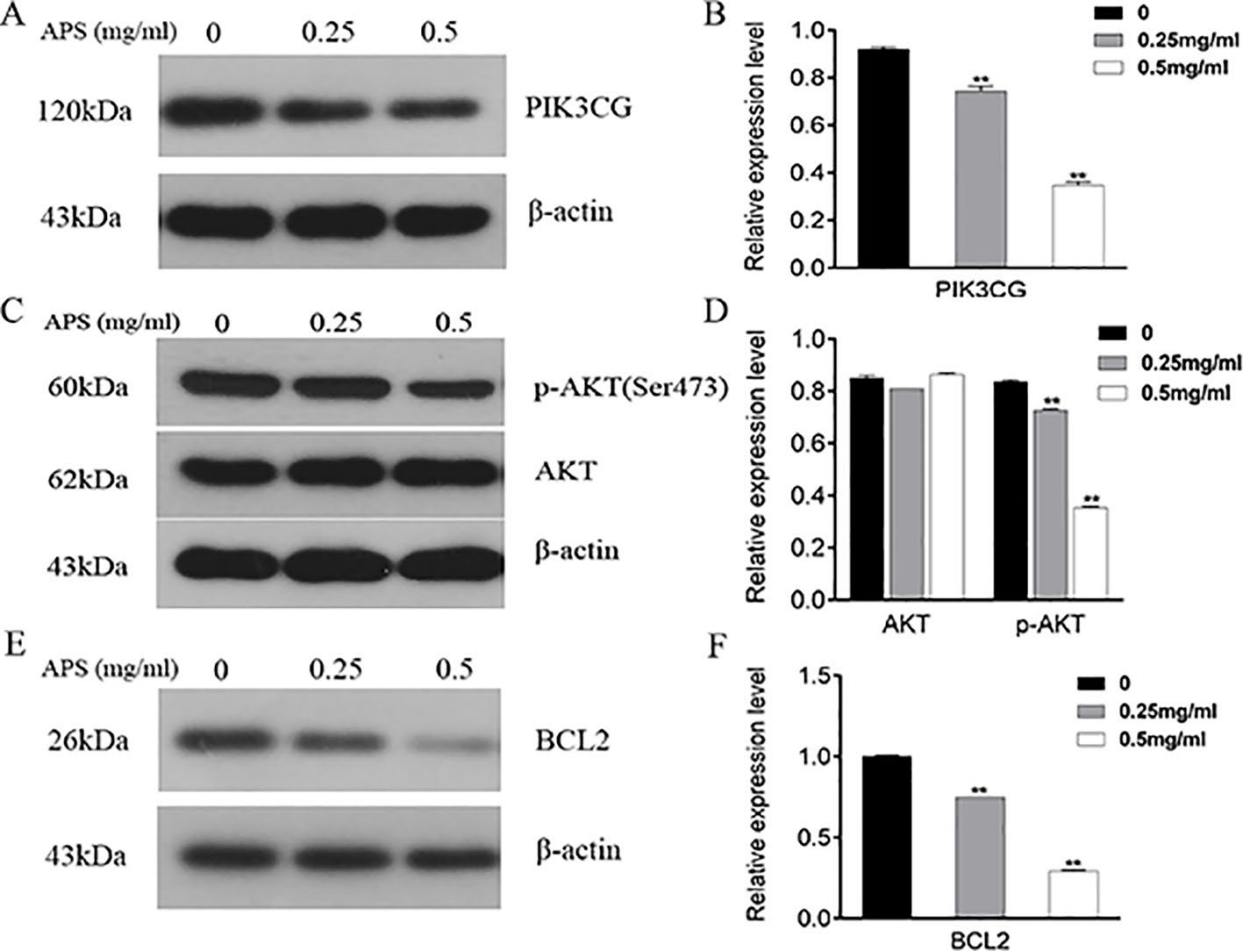
Treatment with APS suppresses the expression levels of PIK3CG/AKT/BCL2-related proteins in MDA-MB-231 cells. Cells were treated in triplicate with or without the indicated concentrations of APS. Then, the protein levels of PIK3CG **(A–B)**, AKT, p-AKT **(C–D)**, and BCL2 **(E–F)** were determined by western blot; β-actin was used as an internal control. Data are expressed as the mean ± SD of each group from three separate experiments. **p <0.01 *vs*. control (0 mg/ml).

As illustrated in **Figure 11**, the proteins associated with cell cycle apoptosis were expressed and detected in the TNBC cell lines by immunoblotting. The key targets PIK3CG, AKT, and BCL2 obtained in this study are closely related to apoptosis. Among them, PIK3CG and AKT are essential regulators of apoptosis, while BCL2 is a key antiapoptotic protein. Therefore, this study aimed to determine whether the apoptotic signaling pathway PIK3CG/AKT/BCL2 is directly affected by APS. The results suggested that APS decreases the expression levels of PIK3CG (**Figures 11A, B**) and BCL2 (**Figures 11E, F**) as the concentration of APS increases. Although there were no significant inhibitory effects on the AKT protein, p-AKT (**Figures 11C, D**) expression was significantly downregulated.

## Discussion

TNBC etiologies manifest as a complex disorganized system (Barabasi et al., 2011), and accumulating evidence has shown that such diseases may not effectively respond to single therapeutic interventions. IP provides hypotheses for the discovery of key compounds and the exploration of their underlying multitarget mechanisms. The study described herein highlights how this approach could provide researchers with completely unintuitive hypotheses for mechanisms of action, thus potentiating innovative findings. Additionally, our case study adds evidence that this method is particularly effective for research on natural products, which are typically multitargeted (Kibble et al., 2015). Here, using IP methods, we developed hypotheses for the effect of the natural product AM on TNBC. A dual CT and CD network was constructed based on the data we excavated, and pathway enrichment and GO BP analyses showed the extensive antitumor activity of effective AM components. The key hub obtained by topology attribute analysis of the network may involve the “drug-target” interaction. Based on the survival analysis of multiple databases and molecular docking, we found the multitargeted intervention of APS, the active ingredient in AM.

Previous studies have shown that APS have many biological activities, including anti-inflammatory, antioxidant, and immunoregulatory properties (Pu et al., 2016; Lv et al., 2017; Zhou et al., 2017; Zhao et al., 2018). Recently, studies have focused on the antitumor activity of APS. Research has shown that APS can effectively reduce cell viability and induce apoptosis in hepatocellular carcinoma (HCC) cells (Huang et al., 2016). APS can also effectively interfere with the autophagy pathway to exert antitumor activity (Zhai et al., 2018). Moreover, the synergistic effect of APS with various chemotherapeutic agents, such as cisplatin and doxorubicin (Li et al., 2008; Kuo et al., 2015; Li et al., 2018a), has also been discovered. Taken together, these studies have shown that APS does have an intervention effect on human solid tumors. However, its effect on the progression of TNBC as well as the underlying regulatory mechanism remains unclear. In our study, MDA-MB-231 cells were used to study the effects of APS on TNBC cells. The results showed a particularly significant intervention activity after APS was applied to TNBC cells, especially at the level of apoptosis. This result is consistent with the widespread enrichment of GO BPs in the “regulation of cell proliferation,” “regulation of cell death,” and “regulation of apoptosis.” Furthermore, based on the analysis of the drug-target synergy network by IP, APS might regulate TNBC by interfering with the PIK3CG/AKT/BCL2 pathway.

Bioinformatics analysis in our study indicates that the expression of PIK3CG, AKT, and BCL2 in TNBC tissues is significantly higher than that in adjacent healthy tissues and in hormone receptor-positive breast tumor tissues. The PIK3CG/AKT/BCL2 pathway plays a key role in the regulation of apoptosis in cells. Among these proteins, PIK3CG and AKT are necessary regulators required for cellular apoptosis. The balance between antiapoptotic proteins (such as BCL2) and proapoptotic proteins affects tumorigenesis and development (Adams and Cory, 2007). Furthermore, molecular docking analysis suggested a good structure fit between APS and the three proteins. Apoptosis is essential for the morphogenesis, development, and homeostasis of multicellular organisms (Majtnerova and Rousar, 2018). Focusing on apoptotic pathways or hubs and disorders caused by inappropriate apoptosis can be a suitable entry point for the treatment of many abnormalities, especially for tumors (Fathi et al., 2018). The experimental results indeed showed that APS at a concentration of 1 mg/ml can effectively promote late apoptosis.

PIK3CG, as a member of the class-I catalytic isoforms of PI3K (Engelman et al., 2006), is a conspicuous target molecule for the regulation of cell survival *via* PI3K pathways and appears to dynamically vary with the expression of PI3K (Kachele et al., 2015). Its illegitimate activation has been proven to be associated with a variety of cancers (Gorlov et al., 2018; Shu et al., 2018; Zhang et al., 2018). Over the past decade, the PI3K signaling pathway has been proven to be one of the most highly mutated systems in human cancers, underscoring its central role in human carcinogenesis (Zhang et al., 2017). The intracellular signaling cascades initiated by or intersecting PI3K are intricate; the most classic of which is the PI3K/AKT signaling pathway. AKT, a serine-threonine kinase downstream of PI3K, is regulated by PI3K. Its overactivation has been proven to stimulate cell cycle progression and promote cell metabolism and invasion resistance (Vivanco and Sawyers, 2002; Carnero et al., 2008). Several subcellular processes mediated by AKT were achieved by phosphorylating different target proteins, including intervention in the BCL2 family. The antiapoptotic BCL2 protein family is often dysregulated in human cancers by a variety of mechanisms. In the process of tumorigenesis and cancer development, cancer cells show dependence on upregulation of the antiapoptotic BCL2 protein family and tend to induce these survival mechanisms (Certo et al., 2006; Juin et al., 2013). Due to their importance in disease biology, especially cancers, the regulatory mechanisms of antiapoptotic BCL2 family members are being extensively studied (Cui and Placzek, 2018). Interestingly, it has been shown that the BCL2 proapoptotic family contributes to the survival-promoting effects of AKT under conditions of cellular stress (Yamaguchi and Wang, 2001; Gardai et al., 2004; Moriishi et al., 2014). Mutually, AKT-mediated phosphorylation of the BCL2 protein abrogates its proapoptotic activity (Jendrossek, 2012; Szymonowicz et al., 2018). Thus, we hypothesized that the PIK3CG/AKT/BCL2 pathway is involved in the interventional regulation of APS in TNBC cells. We found that the expression of PIK3CG, p-AKT, and BCL2 was significantly decreased by increasing APS concentration.

These results also reversely indicate that IP, utilization of the interplay of network pharmacology, big data for disease molecules, and computer-aided simulation, is a convenient way to discover natural products with high efficiency and translation potential. At the same time, IP also provides a system-level approach to understanding multi-target treatment strategies for disease. This also helps to design new molecular frameworks based on intrinsic and extrinsic intervention mechanisms. Natural products have enormous structural diversity and various bioactivities, and the appropriate use of IP methods can initiate new directions and improve our overall understanding.

## Conclusion

In conclusion, by integrated pharmacological methods, we have identified APS as a key product that can interfere with the development of TNBC by disturbing the PIK3CG/AKT/BCL2 pathway. This study further confirmed that APS can effectively inhibit the activity and invasion and enhance the apoptosis of MDA-MB-231 cells as well as exert a key inhibitory effect on the expression of PIK3CG, AKT, and BCL2. Obviously, comprehensive pharmacology is effective as a means of exploring natural products that interfere with cancer.

## Author Contributions

CL, KW, and CS conceived and designed this research. JZ, LL, CG, and KY mined original data and conducted integrated pharmacological analysis. KW, JL, FF and LD performed the experiments and verified the results. CL, HL, and CZ wrote the paper; CS, KW, and LW reviewed and edited the manuscript.

## Funding

This research was supported by the National Natural Science Foundation of China (81473513, 81673799 and 81703915).

## Conflict of Interest

The authors declare that the research was conducted in the absence of any commercial or financial relationships that could be construed as a potential conflict of interest.
