# Supplementary material for: The Modulatory Properties of Astragalus membranaceus Treatment on Triple-Negative Breast Cancer: An Integrated Pharmacological Method
Source: Front Pharmacol. 2019 Oct 14;10:1171. doi: 10.3389/fphar.2019.01171 (PMC6802460; doi:10.3389/fphar.2019.01171)
Supplement: Supplementary file 1 [file Table_1.doc]

| Number | Molecule Name | pubchem ID |
| --- | --- | --- |
| 1 | Mairin | 64971 |
| 2 | Jaranol | 5318869 |
| 3 | hederagenin | 73299 |
| 4 | (3S,8S,9S,10R,13R,14S,17R)-10,13-dimethyl-17-[(2R,5S)-5-propan-2-yloctan-2-yl]-2,3,4,7,8,9,11,12,14,15,16,17-dodecahydro-1H-cyclopenta[a]phenanthren-3-ol | 15976101 |
| 5 | isorhamnetin | 5281654 |
| 6 | 3,9-di-O-methylnissolin | 15689655 |
| 7 | 7-O-methylisomucronulatol | 15689652 |
| 8 | (6aR,11aR)-9,10-dimethoxy-6a,11a-dihydro-6H-benzofurano[3,2-c]chromen-3-ol | 14077830 |
| 9 | Bifendate | 108213 |
| 10 | formononetin | 5280378 |
| 11 | isoflavanone | 160767 |
| 12 | Calycosin | 5280448 |
| 13 | kaempferol | 5280863 |
| 14 | (3R)-3-(2-hydroxy-3,4-dimethoxyphenyl)chroman-7-ol | 10380176 |
| 15 | 1,7-Dihydroxy-3,9-dimethoxy pterocarpene | 5316760 |
| 16 | quercetin | 5280343 |
| 17 | Astragalus polysaccharide | 2782115 |
| 18 | Astragaloside IV | 13943297 |

Table S1. Eighteen active compounds in *Astragalus membranaceus* were identified after ADME and literature screening.
