# Supplementary material for: The Modulatory Properties of Astragalus membranaceus Treatment on Triple-Negative Breast Cancer: An Integrated Pharmacological Method
Source: Front Pharmacol. 2019 Oct 14;10:1171. doi: 10.3389/fphar.2019.01171 (PMC6802460; doi:10.3389/fphar.2019.01171)
Supplement: Supplementary file 3 [file Table_3.doc]

| Table S3. Final compound-related genes obtention | | | | | | | | | |
| --- | --- | --- | --- | --- | --- | --- | --- | --- | --- |
| PGR | PDE3A | ADRB2 | MTND6 | SLC2A4 | NFKBIA | PLAT | E2F2 | FGFR1 | HSD17B1 |
| NOS2 | SLC6A2 | ADRA1D | HSD3B2 | NR1I3 | POR | THBD | ACPP | FGFR2 | RDH8 |
| PTGS1 | CYPS | OPRM1 | HSD3B1 | INSR | ODC1 | SERPINE1 | CTSD | FGFR4 | HMGCR |
| AR | NOS2 | PTGS1 | DPP4 | DIO1 | CASP8 | COL1A1 | IGFBP3 | FGFR3 | CYP51A1 |
| SCN5A | ESR1 | DRD1 | CAMKK2 | PPP3CA | TOP1 | IFNG | IGF | ALOX15B | NR1H2 |
| PTGS2 | PPARG | KCNH2 | AR | PRXC1A | RAF1 | PTEN | CD40LG | ALOX15 | NR1H3 |
| ESR2 | PTPN1 | ESR1 | IKBKB | GSTM1 | SOD1 | IL1A | IRF4 | ADORA1 | CYP17A1 |
| DPP4 | MAPK14 | ADRB1 | AKT | GSTM2 | PRKCA | MPO | ERBB3 | ADORA3 | LDLR |
| HSP90 | GSK3B | F10 | BCL2 | AKR1C3 | HIF1A | TOP2A | PON1 | DYRK1A | VLDLR |
| cdk2 | CDK2 | CHRM5 | BAX | SLPI | RUNX1T1 | NCF1 | PCOLCE | PLG | LRP8 |
| CHEK1 | PIK3CG | ADRA2C | TNF | TRYP1 | ERBB2 | ABCG2 | NPEPPS | NOX4 | SREBF2 |
| PRSS1 | TRYP1 | CHRM4 | AHSA1 | MMP3 | PPARG | GSTP1 | HK2 | LPA | SREBF1 |
| Ncoa2 | PIM1 | OPRD1 | CASP3 | EGFR | ACACA | NFE2L2 | RASA1 | ADORA2A | MAOA |
| CALM | CCNA2 | HTR2A | MAPK8 | VEGFA | HMOX1 | NQO1 | AKR1B10 | MCL1 | MBNL1 |
| PGR | NCOA6 | ADRA1A | MMP1 | CCND1 | CAV1 | PARP1 | POLB | TDP1 | AOX1 |
| CHRM3 | PYGM | SLC6A3 | STAT1 | BCL2L1 | MYC | SLC2A4 | AKR1B15 | ALOX12 | ABCC1 |
| CHRM1 | PPARD | SLC6A4 | cdc2 | FOS | F3 | COL3A1 | AKR1A1 | ALOX12B | ABCC3 |
| GABRA2 | Akr1b1 | MAPK14 | HMOX1 | CDKN1 | GJA1 | CXCL11 | AKR1E2 | ALOXE3 | ABCC2 |
| GABRA3 | Ncoa1 | RXRB | CYP3A4 | EIF6 | IL1B | CXCL2 | SAE1UBA2 | F9 | FYN |
| CHRM2 | F7 | KCNMA1 | CYP1A2 | CASP9 | CCL2 | DCAF5 | MAPT | HSP90AA1 | YES1 |
| ADRA1B | NOS3 | PTGS2 | CYP1A1 | PLAU | PTGER3 | CHEK2 | HSD11B1 | HSP90AB1 | FGR |
| GABRA1 | ACHE | CHRNA7 | ICAM1 | MMP2 | CXCL8 | CLDN4 | HSD11B1L | HSP90B1 | SRC |
| GRIA2 | MAOB | KDR | SELE | MMP9 | PRKCD | PPARA | NR1H4 | PLA2G1B | FRK |
| GABRA6 | RELA | MET | VCAM1 | MAPK1 | BIRC5 | HSF1 | CDC25A | PTPRF | ABCB1 |
| GABRA5 | XDH | TOP2 | NR1I2 | IL10 | DUOX2 | CRP | CDC25B | ACP1 | CA12 |
| IGHG1 | NCF1 | HSP90 | CYP1B1 | EGF | HSPB1 | CXCL10 | TOP2B | PTPRD | CA1 |
| ADH1B | OLR1 | CHEK1 | ALOX5 | RB1 | TGFB1 | CHUK | CRYZ | HSD17B2 | CA2 |
| ADH1C | ADRB1 | JUN | HAS2 | IL6 | SULT1E1 | SPP1 | PTPN2 | HSD11B2 | CDK1 |
| lYSOZ1 | HTR3A | IL4 | GSTP | CDKN2A | MGAM | RUNX2 | FLT1 | CBR1 | CA3 |
| COBT | ADRA2C | SIRT1 | AHR | TP53 | IL2 | RASSF1 | FLT4 | CBR3 | MBNL2 |
| RARA | RXRA | ATP5F1B | PSMD3 | ELK1 | CCNB1 | E2F1 | CYP19A1 | ERBB4 | MBNL3 |
